# Semaphorins and their receptors in pancreatic cancer: Mechanisms and therapeutic opportunities

**DOI:** 10.3389/fonc.2022.1106762

**Published:** 2023-01-13

**Authors:** Dahai Liu, Jie Li, Fei Qi, Hua You

**Affiliations:** ^1^ School of Medicine, Foshan University, Foshan, Guangdong, China; ^2^ Department of Pulmonary and Critical Care Medicine, Peking University Shenzhen Hospital, Shenzhen, China; ^3^ Department of Pediatric Hematology and Oncology, Children’s Hospital of Chongqing Medical University, Chongqing, China; ^4^ Laboratory for Excellence in Systems Biomedicine of Pediatric Oncology, Chongqing Key Laboratory of Pediatrics, Ministry of Education Key Laboratory of Child Development and Disorders, National Clinical Research Center for Child Health and Disorders, Children’s Hospital of Chongqing Medical University, Chongqing, China

**Keywords:** pancreatic cancer, semaphorins, mechanisms, Sema3, Sema4D

## Abstract

Pancreatic cancer (PC) is a malignant tumor with high malignancy that is difficult to diagnose and treat. PC is a major medical problem because of its low early diagnosis rate, high surgical mortality rate, low cure rate, and expensive related testing cost. Therefore, the significance of finding new markers for PC is self-evident. Semaphorins (Semas) have been shown to affect angiogenesis and lymphangiogenesis and can also directly affect the behavior of tumor cells. The expression and related action targets of its family members on PC are summarized in this review.

## 1 Introduction

Pancreatic cancer (PC) is an extremely aggressive, fatal disease caused by early metastatic transmission without curative surgical resection. Most PCs (~90%) are pancreatic ductal adenocarcinoma (PDA) (1). Although the prognosis has improved slightly in recent years, the pancreatic cancer-related mortality rate has gradually increased over the last decade, and the 5-year survival rate is still less than 8% (1, 2). Despite the increasing number of surgical treatments, about 70% of patients will still develop early recurrent (3) within 6–12 months after surgery.

Semaphorins (Semas) have been recognized as critical contributors to neural development (4), the immune response (5, 6), and tumor progression (7). Semaphorins consists of a large family of secreted and membrane-associated proteins with a highly conserved, about 500 amino acids, semaphorin domain. Semaphorins were first mentioned in early 1990s. There are more than 20 types of these proteins have been described as guidance factors assisted axon pathfinding during neuronal development (8, 9). These proteins are divided into eight classes based on structural elements and distribution among different phyla (10). Class 1 and 2 Semas are found only in invertebrates, while class 3–7 are found only in vertebrates. Class 8 members are found in viruses. Class 1, 4, 5, and 6 members are transmembrane Semas. Class 2, 3, and 8 Semas are secreted, and class 7 members are glycosylphosphatidylinositol (GPI)-linked (11).

It was reported that cancer mortality declined by 32% between 1991 and 2019 in the United States. Notably, the risk of cancer death decreased by about 2% per year between 2015 and 2019. The remarkable effects of cancer prevention and control, diagnosis, and treatment in recent years are strongly illustrated by this data (1). The improvement in diagnosis levels has enabled the timely diagnosis of some cancers. Advances in adjuvant chemotherapy, targeted therapy, and combination therapy have significantly improved patient outcomes. Nevertheless, the survival rate of PC is still low. Therefore, we summarized the current relevant mechanisms of Semas in PC and their potential for PC treatment in this review.

## 2 Sema3

### 2.1 Sema3A

Sema3A has received less focus than its receptors. The study of Sema3A and its receptors, such as plexinsA1-A4 (PLXNA1-A4) and neuropilin-1 (NRP1) in PC, demonstrates that SEMA3A had no impact on the growth or survival of pancreatic tumor cells and was upregulated in PC. Its signaling might partially mediated by Rac1 (12). Interestingly, one research study composed a novel type of hybrid peptide, Sema3A-lytic, which is be formed of two functional amino acid domains: a sequence that binds to NRP1 from Sema3A and a cytotoxic lytic peptide. In addition, this hybrid peptide shows that the Sema3A-lytic hybrid peptide would be a possible anti-cancer agent for treating human PC (13). This may provide a new idea for subsequent studies.

### 2.2 Sema3B

Sema3B, as a potential tumor suppressor gene, gene loss or down-regulation of its function can promote tumor progressions, such as in lung cancer, breast cancer, ovarian cancer, liver cancer, and cholangiocarcinoma (14–17). Plexins are essential receptors for Sema3B activation of downstream signaling pathways, and Sema3B function depends on the NRP receptor to provide binding sites for both receptors. This can exert anti-tumor angiogenesis and promote tumor cell apoptosis through specific binding to the high affinity of both receptors. Sema3B can compete with VEGF for NRP receptors, hindering the promoting effect of VEGF on vascular endothelial cell division, and partly inhibiting angiogenesis. Thus, it indirectly blocks the nutrient supply of tumor cells and slows down cell division (18). Gao (18) et al. shows that lentiviral vector transfection can construct a stable Sema3B upregulated model in PC CFPAC-1 and PANC-1 cell lines. Overexpression of Sema3B could significantly inhibit the proliferation, invasion, and migration of CFPAC-1 and PANC-1 cells, which means that Sema3B may have an essential role in inhibiting tumor genesis and development in PC. The expression level of Sema3B in PC tissues is closely related to the PC tumor stage, local invasion, lymphatic metastasis, and distant metastasis, and the expression level of Sema3B in PC tissues is closely related to the degree of malignancy of PC. The lower the Sema3B expression level, the higher the pathological malignancy of PC. However, due to the few studies on Sema3B in PC, its specific molecular mechanism still needs to be elucidated.

### 2.3 Sema3C

Sema3C has a role in promoting tumor development (19), and it is hypomethylated, and its expression increases with tumor progression (20) in PC. Xu (21) et al. suggested that abnormal expression of Sema3C is associated with poor prognosis in patients with PDAC, and high Sema3C expression in PC was associated with a shorter survival time (22). Sema3C may promote PC cell migration and invasion by inducing the epithelial-mesenchymal transition (EMT) and may contribute to cancer therapy. The extracellular signal-regulated kinase (ERK)1/2 is the most critical pathway for promoting cell proliferation, metastasis, and EMT during tumorigenesis. Sema3C promoted the phosphorylation of ERK in PC and confirmed that Sema3C mediated cell proliferation in PC both *in vitro* and *in vivo*. Furthermore, Proliferation, migration, invasion, and EMT in a PC cell line attenuated and PDAC cell tumorigenesis upon after xenotransplantation into nude mice reduced since Sema3C was knockdown. Overexpression of Sema3C had the contrary effects and boosted the ERK1/2 signaling pathway (21). Furthermore, the combination of trametinib (MEK inhibitor, downstream of KRAS) and Sema3C inhibitor can induce a synergistic effect in KRAS G12D cells, which shows Sema3C might be a potentially prospects and attractive target for PC therapy, especially in patients with G12D mutation in KRAS (22). These features suggest the strong potential of Sema3C in the treatment of PC.

### 2.4 Sema3D

Sema3D and its receptor PlexinD1 (PlxnD1) are particularly interesting, as they are the part that is frequently amplified and mutated in human PDA. Jurcak (23) et al. found that the invasive and metastatic capacity of PDA cells is increased due to the interaction of Sema3D with its co-receptors PlxnD1 and Neuropilin-1. It is supported in another study (24) that tumor cells expressing Sema3D can travel toward PlxnD1-expressing neurons, according to their Diagnosis Related Groups (DRG) in vitro data. However, the molecular mechanism of Sema3D in assisting increased PDA cell invasion and metastasis is unknown. Interestingly, this increase of glycolytic gene expression was inhibited with the neutralizing antibody PlexinD1. They also suggested that Sema3D signaling pathway could offer another selectivity in mutant Kras cells.

Moreover, Sema3D in PC is interesting in the conduction relationship between Sema3D, Annexin A2 (AnxA2), and PlexinD1. Foley (25) et al. elucidated one mechanism of PDA metastasis formation: Sema3D can bind to PlxnD1 on the surface of PDA tumor cells in the extracellular space by the regulation of its autocrine function control of AnxA2. This mechanism has also been proven to promote the invasion and metastasis of PDA cells (26) and the tumor. Mice carrying PDA prolong survival and reduces metastasis due the knockdown of Sema3D (24). These studies increase therapeutic options in PDA and provide a strong rationale for the development of adjuvant therapies targeting AnxA2 and Sema3D for PDA after local resection (26).

### 2.5 Sema3E

**Figure 1:**
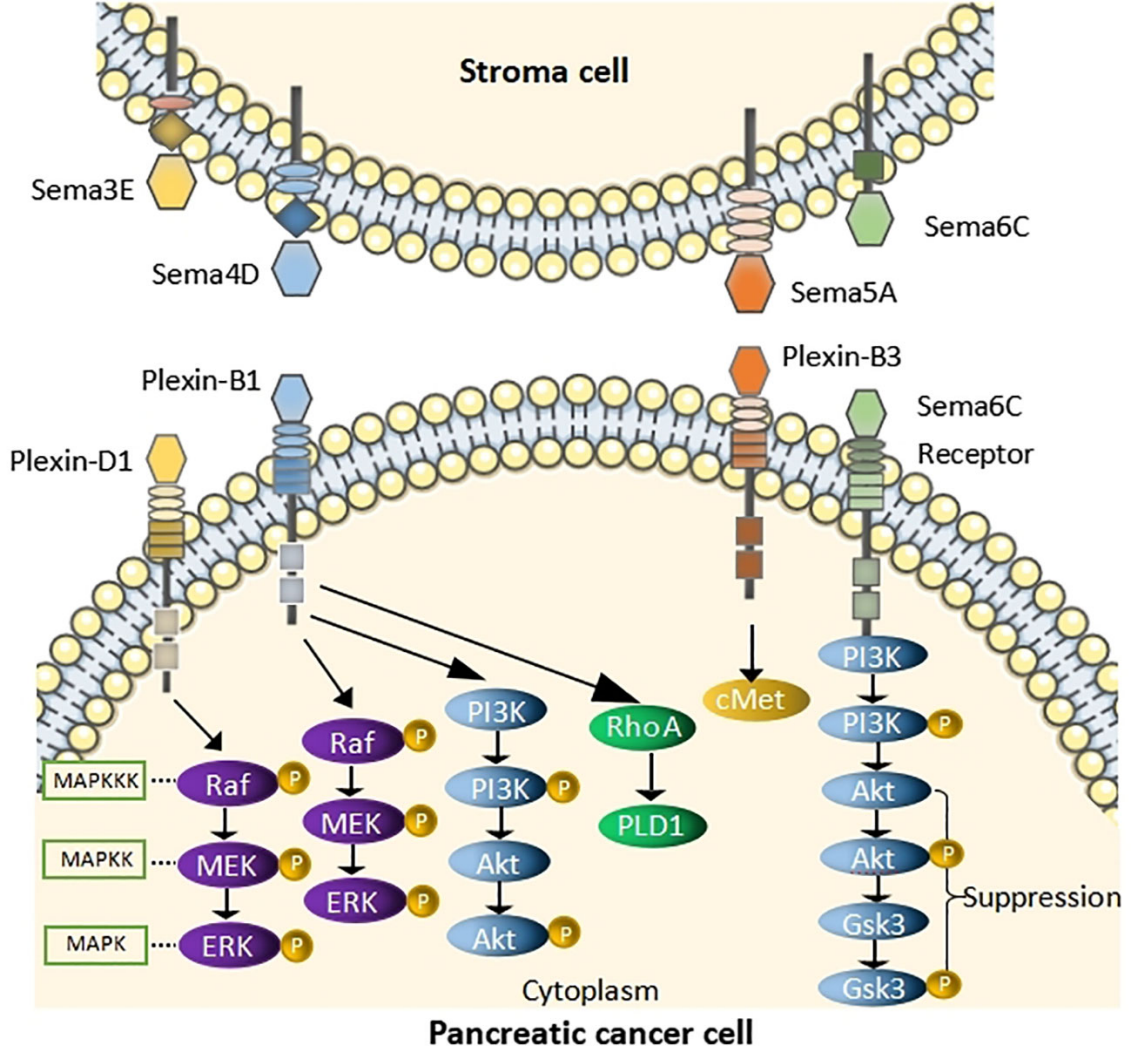
Schematic of pathway depicting the role of Semas in PC Sema3E binds to Plexin-D1and induces cell proliferation by acting through the MAPK/ERK pathway. Sema4D binds to Plexin-B1, activates RhoA, causes the phosphorylation of MAPK and Akt. Sema5A binding to Plexin-B3 activates cMet signaling to enhance migration of PC cells. Sema6C binds to its receptor and suppress AKT/GSK3 signaling pathway.

Unlike the other three classes of signaling elements, Sema3E can directly bind to its receptor, PlxnD1, which activates cellular signaling. This combination has been shown to contribute to the aggressive and metastatic spread of cancer cells. Lin et al. have shown that Sema3E was significantly expressed in the nuclei of PC cells and overexpressed in human PC which was associated with tumor progression and poor survival (27, 28). Also, they have shown that overexpression of Sema3E in PC cells promoted cell proliferation and migration *in vitro*, while cancer cell proliferation and migration *in vitro* had the opposite function (27). In other words, higher incidence and growth of tumors were exhibited by Sema3E overexpressed cells (28), whereas they were reduced by Sema3E knockout cells. Moreover, Sema3E induces cell proliferation through MAPK/ERK pathway (28) (**Figure 1**). Therefore, Sema3E may be a suitable prognostic marker and an attractive new therapeutic target. It has been confirmed in a study (29) that Sema3E is significantly upregulated after therapeutic expression of panc28 cell lines and is one of the common biomarkers.

### 2.6 Sema3G

Interestingly, the ADAMTS9-AS1-SEMA3G pathway is predicted to play an essential role in immune cell regulation by database screening and validation (30). TCGA and GEO databases were analyzed to screen differential lncRNA ADAMTS9-AS1 associated with prognosis. GO and KEGG enrichment analyses were performed, and the results showed that the gene was significantly associated with immunity. The infiltration, significant positive correlation with B cells, CD4^+^ T cells, CD8^+^ T cells, neutrophils, macrophages, and dendritic cells (DCs) was verified using the TIMER database, and a more significant correlation with CD8^+^ T cells and macrophages in PC. Negative correlation between the lncRNA and tumor grade was shown in a clinical correlation analysis. Thus, it shows that the higher the lncRNA expression in PC, the more immune infiltration in the tumor, the lower the tumor grade, and the better the patient prognosis (30). Therefore, the ADAMTS9-AS1-SEMA3G pathway is predicted to play an essential role in immune cell regulation.

Additionally, Gao (31) et al. have proven the role of Sema3G in proliferative capacity and cell migration in PC cells. Human PC PANC-1 cells were transfected with the Sema3G lentivirus interference vector, whose proliferation, invasion, and migration capacity were significantly decreased, indicating that Sema3G could significantly inhibit the proliferation, invasion, and migration of PC. The mechanism of Sema3G inhibiting PC invasion and metastasis and the signaling pathways it works with to inhibit PC progression still needs further intensive investigation.

### 2.7 Sema4D

Sema4D has been one of the research hotspots in recent years. It has been deemed to promote the progress of malignancies by affecting cell proliferation, apoptosis, and immigration (32). Sema4D can bind to the ligand Plexin-B1 and activate small GTPase Ras homolog gene family, member A (RhoA), causing the phosphorylation of MAPK and Akt, thereby enhancing the invasive energy of PC cells (33, 34) (**Figure 1**). It was reported in the relevant literature that inhibition of Sema4D in tumor cells not only inhibits tumor angiogenesis but also may restore T-cell immune activity specific to antigens (34, 35). Notably, Sema4D-siRNA downregulates the expression of Sema4D in pancreatic cells, inhibiting the proliferation of PC cells and reducing their invasive ability and apoptosis (36). Therefore, a team proposed immunotherapy strategies to block Sema4D, which could improve the sensitivity of PDAC to immune checkpoint blockade (ICB) (37), and treatment with Sema4D blocking antibody improved response to ICB in combination by using the standard of care FOLFIRINOX in preclinical murine studies (38). Moreover, the considerable progress of Pepinemab, the Sema 4D antibody, is well tolerated and has potent anti-tumor activity (39), but resistance to immunotherapy often limits patient efficacy. It has been suggested in studies that when Sema4D antibodies are combined with immune checkpoint inhibitors, such as the anti-Sema4D antibody Pepinemab, and combined with the immune checkpoint inhibitor PD-L1 mAb Avelumab, they can enhance T-cell activity, thus persistently inhibiting tumor growth (40).

### 2.8 Sema5A

Sema5A is tumor-promoting in PC. Moreover, Sema5A is expressed in most PC tissues and is not or low expressed in normal pancreatic tissues, which elucidates that Sema5A could be a marker for PC (41, 42). Furthermore, upregulation of mouse Sema5A in Panc1 enhanced tumor cell proliferation and the incidence of distant metastasis to the lymph nodes, liver, spleen, and peritoneal cavity after in situ injection in mice. It was suggested by these results that Sema5A was associated with tumor growth, metastatic potential, and invasiveness (43). It is noteworthy that PC cell migration is enhanced by Sema5A by activating cMet signaling in a PlexinB3-dependent manner (44) (**Figure 1**). Additionally, proliferation and angiogenesis are promoted by SEMA5A in the primary tumor setting, thereby enhancing metastasis (44, 45). Thus, Sema5A/PlexinB3 may represent an attractive targetable axis in PC.

### 2.9 Sema6C

Sema6C might be a potential tumor suppressor in PC and serve as a poor prognostic biomarker in PC (45). Sema6C, a tumor suppressor in PC, causes reduction of cyclin D1 expression and cell proliferation which includes cyclin-dependent kinase 4/6(CDK4/6) (46) by inhibiting the AKT/GSK3 signaling axis (47) (**Figure 1**). Moreover, they demonstrated a brand new regulatory role of miR-124-3p in suppressing Sema6C and suggested the treatment of Sema6C-downregulated cancer by CDK4/6 inhibitors (48).

## 3 Potential application

Recently, Semas have been aberrantly expressed in many tumors and have a regulatory role in tumor development, represented by Sema3 and Sema4. An anti-Sema3A antibody has been patented to reduce immunosuppression caused by tumor-secreted Sema3A, and is available to the treatment of Alzheimer disease and immune dysfunction; Sema4D inhibitors are also patented to increase the frequency of tumor-infiltrating leukocytes by blocking the combination of Sema4D with its receptor (49), and is suitable for the treatment of human head and neck cancer, colon cancer, breast cancer, etc (50). Moreover, in Phase I prospective multiple escalating dose trials completed in patients with advanced refractory solid tumors, treatment with anti-Sema4D antibodies remains well tolerated (51) and still be safe, such as the combination of Avelumab and the Sema4D inhibitor Pepinemab (52). Semas, which may be used as prognostic markers, can be further investigated to select molecular inhibitors with high selectivity and establish PDX models for drug selection evaluation. In addition, signaling pathways that have not yet been identified can be screened and validated against databases to obtain relevant signaling pathways, on which further studies can be conducted.The current research on Semaphorins in PC is only singularly focused on one or two class, and it is hoped that some investigators will keep further research on the above viable and relevant targets to enhance pre-prognostic and prognostic diagnosis, the addition of synthetic emerging drugs and novel combinations of existing drugs.

## 4 Conclusions and future perspectives

The targets and treatment of Semaphorins in PC are shown in **Table 1**. Further research is needed to investigate the mechanism of Semas in PC, with the most promising potential being Sema3C and Sema4D, which are already research hotspots. Among them, the Sema5A/plexinB1 axis and the 6CSEMA6C AKT/GSK3 axis provide us with brand-new ideas, but related research still needs to elucidate its practical feasibility further. The treatment related to Sema4D shows that the multicombination of drug treatment can solve the problem of drug resistance, improve the durability of the treatment, and make noteworthy progress in treating PC.

**Table 1:** Related targets and treatment of semaphorins in pancreatic cancer.

Semaphorins	Function	Target/Therapy	Reference
3A	upregulate	Sema3A/PLXNA1-A4&NRP1 does not affect the growth or survival of pancreatic tumor cells but upregulated PC	(12, 13)
		Sema3A-lytic hybrid peptide may be the treatment of human pancreatic cancer	
3B		Lentiviral vector transfection can construct a stable Sema3B upregulated model in PC CFPAC-1 and PANC-1 cell lines; Overexpression of Sema3B could significantly inhibit the proliferation, invasion, and migration of CFPAC-1 and PANC-1 cells	(18)
3C	promote	Promote tumor growth and metastasis in PC by activating the ERK 1/2 signaling pathway	(21, 22)
	suppress	Sema3C inhibition sensitizes KRAS or MEK1/2 inhibition in PC cells	
		The combination of trametinib and Sema3C inhibitor can induce a synergistic effect in KRAS G12D cells	
3D	promote	Sema3D/AnxA2/PlxnD1 promotes the invasion and metastasis of PDA cells and tumor	(21)
3E	promote and suppress	Overexpression of Sema3E in PC cells promoted cell proliferation and migration *in vitro* Sema3E knockout cells suppressed cancer cell proliferation and migration *in vitro*	(27, 28)
		Sema3E induces cell proliferation by acting through the MAPK/ERK pathway	
3G	suppress	Sema3G lentiviral vectors inhibiting cell proliferation, invasion, and migration	(30)
4D	enhance	Sema4D/Plexin-B1: Activate A-Ras, cause the phosphorylation of MAPK and Akt, enhance the invasive energy	(32–34)
	downregulate	Sema4D-siRNA downregulates Sema4D expression in PC	
5A	activate and enhance	SEMA5A/PlexinB3 axis: enhance PC cell migration by activating cMet signaling in a PlexinB3-dependent manner	(43, 44)
6C	suppress	SEMA6C/AKT/GSK3 axis: suppresses cell proliferation	(46)

## Author contributions

HY designed the study and contributed to manuscript revision. DL, JL and FQ collected the data, interpreted the data, and wrote the draft of the manuscript. All authors contributed to the article and approved the submitted version.
